# A Disorder of Sex Development in a Holstein–Friesian Heifer with a Rare Mosaicism (60,XX/90,XXY): A Genetic, Anatomical, and Histological Study

**DOI:** 10.3390/ani11020285

**Published:** 2021-01-23

**Authors:** Izabela Szczerbal, Marcin Komosa, Joanna Nowacka-Woszuk, Tomasz Uzar, Marek Houszka, Jerzy Semrau, Magdalena Musial, Michal Barczykowski, Anna Lukomska, Marek Switonski

**Affiliations:** 1 Department of Genetics and Animal Breeding, Poznan University of Life Sciences, 60-637 Poznan, Poland; izabela.szczerbal@up.poznan.pl (I.S.); joanna.nowacka-woszuk@up.poznan.pl (J.N.-W.); 2 Department of Animal Anatomy, Poznan University of Life Sciences, 60-625 Poznan, Poland; marcin.komosa@up.poznan.pl (M.K.); tomasz.uzar@up.poznan.pl (T.U.); 3 Department of Preclinical Sciences and Infectious Diseases, Poznan University of Life Sciences, 60-637 Poznan, Poland; marek.houszka@up.poznan.pl (M.H.); anna.lukomska@up.poznan.pl (A.L.); 4 Center for Animal Health and Reproduction, 88-200 Radziejow, Poland; jassemrau@gmail.com (J.S.); madzix.es@gmail.com (M.M.); m.barczykowski@op.pl (M.B.)

**Keywords:** 2n/3n, cattle, diploid/triploid, mixoploidy, intersexuality, mosaicism

## Abstract

**Simple Summary:**

Disorders of sex development (DSDs) are congenital conditions in which a discordance between chromosomal, gonadal, or phenotypic sex is observed. DSDs are serious problems in animal breeding, as they lead to sterility. In cattle, the most common form of DSD is freemartinism, which manifests as the presence of leukocyte chimerism (XX/XY), and occurs in heifers originating from heterosexual twin pregnancy. Other forms of DSD are rarely observed in this species. In this study, we describe a very rare diploid/triploid (60,XX/90,XXY) condition in a DSD heifer. Comprehensive clinical, anatomical, histopathological and genetic analysis was performed.

**Abstract:**

In this study, we describe an eighteen-month-old Holstein–Friesian heifer with a deformed vulva, located abdominally. The heifer showed typical signs of estrus. A comprehensive anatomical and histopathological examination revealed a blind-ended vagina and an additional section of urethra, which became a part of the shortened penis. Cytogenetic analysis showed the presence of two cell lines: 60,XX and 90,XXY. The frequency of the triploid cell line was low (3%) in leukocytes and elevated (35%) in fibroblasts. The molecular detection of Y-linked genes (*SRY* and *AMELY*) in the blood, skin, hair follicles, and buccal epithelial cells confirmed the presence of a cell line carrying the Y chromosome. Genotyping of 16 microsatellite markers in DNA isolated from hair follicles and fibroblast culture showed the presence of one (homozygous) or two variants (heterozygous) at all the studied loci, and allowed chimerism to be excluded. We concluded that the heifer had diploid/triploid (60,XX/90,XXY) mosaicism. To our knowledge, this is only the fifth such case to be reported worldwide in this species. Since cytogenetic studies are routinely performed on in vitro cultured leukocytes, we suspect that the prevalence of this chromosome abnormality is underestimated, as it is known from published reports that the frequency of the triploid cell line is usually very low in leukocytes.

## 1. Introduction

Disorders of sex development (DSDs) are classified into three main categories: sex chromosome DSDs, XX DSDs, and XY DSDs. Abnormal sex chromosome constitutions are well documented in domestic animals [1,2,3]. Freemartinism is the most common type of DSD in cattle. It occurs in heifers originating from heterosexual twins, and is characterized by the presence of XX and XY cell lines (XX/XY leukocyte chimerism). The affected females are sterile due to their underdeveloped reproductive systems. In dairy cattle breeds, an increased trend of twin pregnancies is observed, and this type of DSD is thus considered a serious health and breeding problem [4,5].

Diploid/triploid (2n/3n) mixoploidy is a unique chromosome abnormality in mammals. To date, several such cases have been reported in domestic animals, including cattle [6,7,8], cats [9,10], and horses [11]. In humans, over eighty such patients have been diagnosed [12]. Interestingly, disorders of sex development have been observed in patients with 46,XX/69,XXY mixoploidy.

Mixoploidies (2n/3n) can occur in a form of mosaicism (where cell lines originate from a single zygote) or chimerism (where the lines are derived from different zygotes), and the additional genome can have a parental or maternal origin. Mixoploidy can be caused by: (a) delayed extrusion of the second polar body, followed by aberrant segregation of the entire parental genome into blastomeres during early embryonic mitoses; (b) fertilization by two sperms, followed by the fusion of one male pronucleus with the female pronucleus in a zygote and delayed fusion of the second male pronucleus with diploid nucleus of a daughter blastomere; or (c) the fusion of diploid and triploid embryos [12]. It is worth mentioning that cytogenetic analysis of mixoploid human patients, on the basis of in vitro cultured leukocytes, has usually revealed a normal diploid karyotype or a very low proportion of triploid metaphase spreads. On the other hand, the incidence of the triploid cell line has been found to be elevated in in vitro cultured fibroblasts [13,14].

We present here a comprehensive analysis of a new case of diploid/triploid mosaicism in a Holstein–Friesian heifer with a disorder of sex development.

## 2. Material and Methods

### 2.1. Case Presentation

**Figure 1:**
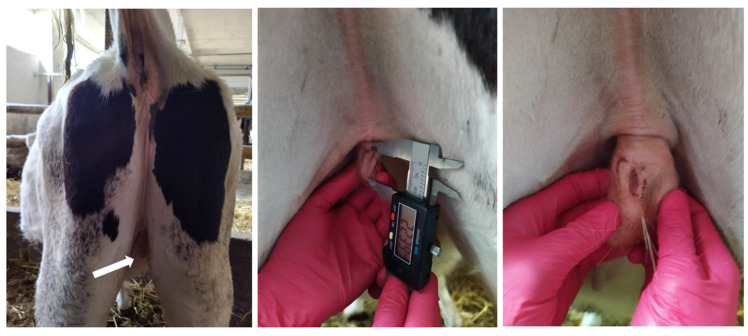
External genitalia of the studied heifer. The deformed vulva is indicated by an arrow.

An eighteen-month-old Holstein–Friesian heifer was subjected to clinical examination due to abnormal development of the external genitalia (Figure 1). The vulva, located abdominally, had a small clitoris and underdeveloped labia. The opening of the urethra was palpated inside the vulva. The permeability of the vagina, approximately 30 cm in length, was observed by inserting an insemination catheter. Rectal examination revealed a normally developed uterus and gonads resembling ovaries. The heifer displayed typical heat symptoms, such as bellowing, restlessness, and trailing. Ultrasound examinations revealed active ovaries with a large ovarian follicle (approximate diameter 17 mm). Concentrations of steroid hormones in serum were as follows: 0.04 µg/L (estradiol) and 0.07 µg/L (testosterone).

The heifer was barren due to the observed abnormalities and all tissue samples for further analysis were collected postmortem at a local slaughterhouse.

### 2.2. Anatomical and Histopathological Studies

In postmortem examination, the reproductive and urinary organs were removed and fixed in neutral 10% formalin solution. Individual sections of the urogenital apparatus were prepared and macroscopically described. Samples of tissues were then taken from the ovaries, fallopian tubes, uterine horns, uterine body, cervix, vagina, ureters, and urethra. Paraffin sections (3 μm), stained with hematoxylin and eosin (H&E), were examined, and micrographs were taken using an Axio Lab.1 microscope (Carl Zeiss, Oberkochen, Germany) equipped with an ERc5s digital camera (Carl Zeiss, Oberkochen, Germany). Zen 2.3 software (blue edition; Carl Zeiss Microscopy, 2011) were used.

### 2.3. Cytogenetic Analysis

Chromosome preparations were obtained from in vitro leukocyte and fibroblast cultures. The cells were cultured using RPMI-1640 medium (Sigma-Aldrich Inc., St. Louis, MO, USA) or Dulbecco’s modified Eagle’s medium (DMEM) (Sigma-Aldrich Inc., St. Louis, MO, USA), supplemented with 15% (*v*/*v*) fetal calf serum, and 1% (*v*/*v*) penicillin/streptomycin and phytohemagglutinin (in case of leukocyte culture) at 37 °C in a humidified atmosphere of 5% CO_2_. After colcemid treatment, the standard harvesting procedure with hypotonic and fixative steps was used. Chromosomes were analyzed using Giemsa, DAPI (4’,6-diamidino-2-phenylindole), and C-band staining techniques. Bovine sex chromosomes were identified based on their bi-armed morphology and characteristic banding pattern after C-banding. The slides were examined with an epifluorescence Nikon E600 Eclipse microscope ( (Melville, NY, USA) equipped with a cooled CCD digital camera (Melville, NY, USA) and Lucia software (Laboratory Imaging, Ltd., Prague, Czech Republic).

### 2.4. Molecular Analysis

Genomic DNA was isolated from different tissues (blood, skin, buccal epithelial cells, and hair follicles) with the use of commercial kits (A&A Biotechnology, Gdynia, Poland). A polymerase chain reaction (PCR) was used to detect the Y-linked *SRY* gene, using primers that have previously been described [15]. The PCR conditions were: initial denaturation at 95 °C for 5 min, 35 cycles of denaturation at 95 °C for 30 s, annealing of primers at 60 °C for 30 s, elongation at 72 °C for 40 s, and final elongation at 72 °C for 5 min. The presence of the amplicon (813 bp) was checked with the use of agarose gel electrophoresis under standard conditions.

The highly sensitive droplet digital PCR (ddPCR) technique was used to detect the copy number of *AMELX* and *AMELY* genes. The primer and probe sequences were as described earlier [4]. The reaction mixture contained 10 µL of 2 × ddPCR Supermix for Probes (Bio-Rad, Hercules, CA, USA), 1 µL of 20 × primers/FAM (6-carboxyfluorescein) probe, 1 µL of 20 × primers/HEX (hexa-chloro-fluorescein) probe (900 nM primers and 250 nM probes), 1 µL of BsuI restriction enzyme (diluted 1:2), and 20 ng of DNA. The PCR reaction mixtures were partitioned into approximately 20,000 droplets using a QX200 droplet generator (Bio-Rad, Hercules, CA, USA). PCR was performed on a Gradient T100 Thermal Cycler (Bio-Rad, Hercules, CA, USA) using the following thermal cycle conditions: denaturation at 95 °C for 10 min; 40 cycles at 94 °C for 30 s and 55 °C for 60 s (ramp rate 2 °C/s); 98 °C for 10 min, and 10 °C until reading time. The droplets were analyzed on a QX200 droplet reader (Bio-Rad, Hercules, CA, USA). The concentration of *AMELX* and *AMELY* genes was calculated using the Poisson distribution with Quantasoft software (Bio-Rad, Hercules, CA, USA).

A set of sixteen microsatellite markers, commonly used for parentage control, were analyzed at Dr. van Haeringen Laboratorium (VHL Genetics, Wageningen, The Netherlands). The analysis was carried out on DNA samples isolated from hair follicles and fibroblast culture of the DSD heifer, as well as from frozen semen doses of the sire.

## 3. Results

Detailed anatomical and histopathological studies of the heifer revealed several developmental abnormalities. The internal female genitalia—the ovaries, fallopian tubes, and uterus—presented their regular anatomical and histological structures. The ovaries had a normal structure. In the left ovary, several vesicular ovarian follicles, corpuses albicans (white bodies), and a large corpus luteum (yellow body) occupying approx. 2/3 of the ovary’s surface, were visible. In the right ovary, a single Graafian follicle and several small vesicular follicles, as well as white bodies were observed.

**Figure 2:**
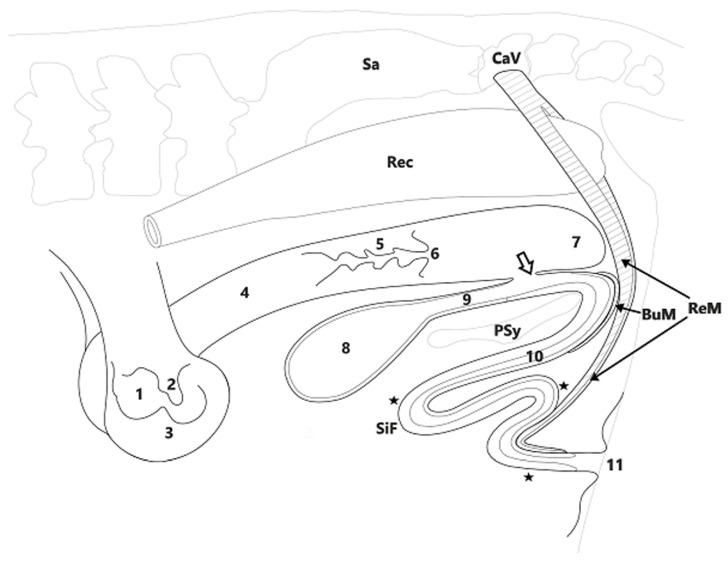
The urogenital system of the studied heifer. Abbreviations: Sa: sacral bone; CaV: caudal vertebrae; Rec: rectum; PSy: pelvic symphysis; BuM: bulbospongiosus muscle; ReM: retractor muscle of penis; SiF: sigmoid flexure; 1: ovary; 2: fallopian tube; 3: horn of uterus; 4: body of uterus; 5: uterine cervix; 6: external orifice of uterus; 7: blind-ended vestibule of the vagina; 8: urinary bladder; 9: female urethra; 10: male urethra included in penis; 11: external urethral orifice and deformed vulva. White arrow: the beginning of male urethra; asterisks: histological evaluation sites.

Macroscopic changes were seen only in the structure of the vagina and in subsequent parts of the urogenital apparatus. Although the external orifice of the uterus and the fornix of vagina were properly formed, the vestibule of the vagina ended blindly. The urethra, even though it was in contact with the vagina, thus extended and ran further towards the ischiatic arch, and then turned towards the ventral side. It resembled a sigmoid flexure under the pelvic symphysis, as is typical in bulls (Figure 2).

**Figure 3:**
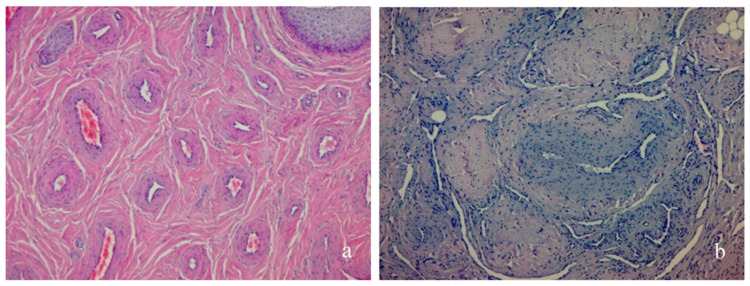
Histological features of the urethra: (**a**) spongial body with irregular blood vessels in fibrous collagen stroma; (**b**) cavernous body with helicine arteries and irregular slit-like vessels alternating with branches of coarse fibrin collagen, hematoxylin and eosin (H&E) staining.

The urethra was lined with stratified or pseudostratified cylindrical epithelium, which in the distal part becomes a stratified squamous epithelium. In the spongial body, numerous irregular blood vessels were seen in the fibrous collagen stroma (Figure 3a). The ovaries, urethra, the spongial body, and the individual columns of the cavernous body had typical thick-wall helicine arteries and irregular slit-like vessels, alternating with branches of coarse fibrin collagen (Figure 3b).

The whole structure resembled a penis, though shortened. This is also confirmed by the presence of muscles that would accompany the male genital organs. In the proximal segment, the bulbospongiosus muscle covering the penis was located. Under this muscle was located the penile bulb. A penile retractor muscle could also be distinguished; this had two bellies and ended in the distal part of the penis. The glans penis and prepuce were absent. Moreover, no accessory genital glands typical of males were noted.

**Figure 4:**
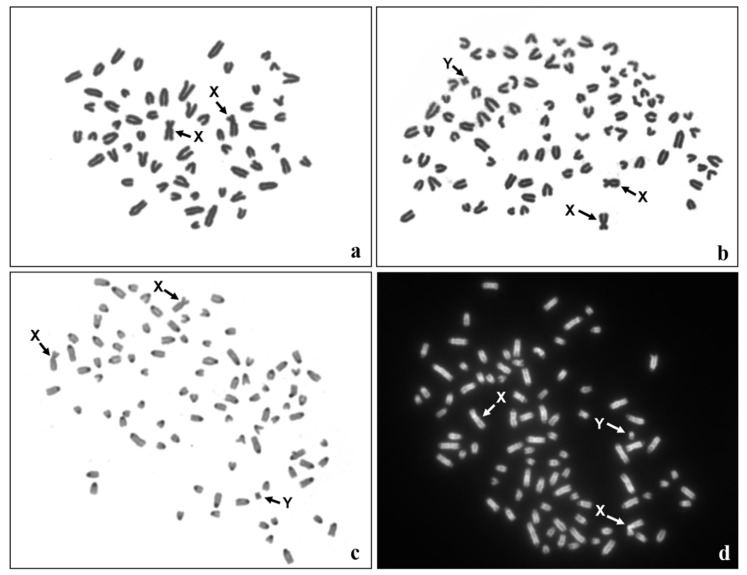
Metaphase spreads originating from the in vitro fibroblast culture: (**a**) diploid metaphase (60,XX), Giemsa staining; (**b**) triploid metaphase (90,XXY), Giemsa staining; (**c**) triploid metaphase (90,XXY), C-banding and (**d**) triploid metaphase (90,XXY), DAPI-banding. Sex chromosomes are indicated by arrows.

Cytogenetic evaluation of Giemsa-stained metaphase spreads (n = 400) derived from the lymphocyte culture revealed a diploid chromosome number (60,XX) in 97% of cells, while in 3% of cells a triploid set of chromosomes (90,XXY) was observed (Table 1). Additional analysis of 200 metaphase spreads originating from the fibroblast culture also showed the presence of two cell lines: 60,XX (65% of the spreads) and 90,XXY (35% of the spreads) (Table 1, Figure 4a,b). DAPI and C-banding techniques confirmed the normal morphology of sex chromosomes (Figure 4c,d). Taking these results together, mixoploidy (60,XX/90,XXY) was identified in the heifer.

**Figure 5:**
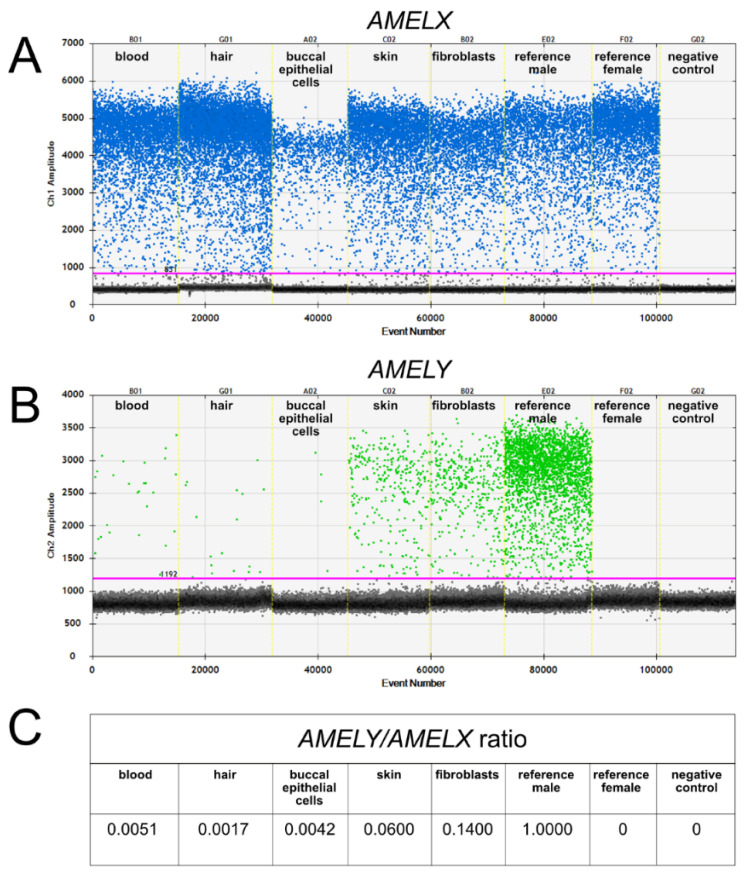
Detection of *AMELX* (**A**)*, AMELY* (**B**) genes by droplet digital polymerase chain reaction (ddPCR) and the ratio between number of copies, *AMELY/AMELX* (**C**).

To confirm our cytogenetic observation of the 90,XXY cell line, we molecularly detected an X-linked gene (*AMELX*) and two Y-linked genes (*SRY* and *AMELY*). Classic PCR revealed the presence of the *SRY* gene in blood, skin, fibroblast culture, and buccal epithelial cells, but not hair follicles (Figure S1, Table 1). Highly sensitive ddPCR detected the *AMELX and AMELY* genes in all samples—that is, in blood, skin, fibroblast culture, buccal epithelial cells, and hair follicles (Figure 5 and Table 1). The *AMELY/AMELX* ratio in the heifer was low in the hair follicles (0.0017) and buccal epithelial cells (0.0042), but higher in the skin (0.06) and fibroblast culture (0.14). These results showed that Y-linked genes were present in all the samples, but that the 60,XX cell line was predominant.

Genotyping of DNA samples derived from the fibroblast culture and hair follicles of the heifer was performed with the use of sixteen microsatellite markers. The same genotype was observed at all loci. In addition, the genotyping of semen samples of the sire showed that the variant inherited from the sire was present at all loci in samples derived from the DSD heifer (Table S1). These results indicate that the cell lines found in the heifer originated from a single zygote. This means that the heifer was a case of diploid/triploid (60,XX/90,XXY) mosaicism.

## 4. Discussion

Ambiguous external genitalia, such as an enlarged clitoris, increased anovulval distance, and the presence of long vulval hair, are occasionally observed in freemartins, while abnormal development of the internal genital tract (short vagina, absence of cervix, hypoplastic uterus) are common in such animals [16,17]. An increased anovulval distance was seen in the DSD heifer presented here. Our preliminary assumption was thus that this animal represented a case of freemartinism. However, postmortem anatomical and histological studies revealed other abnormalities, including a blind vagina and an extended urethra surrounded by tissues typical of a rudimentary penis. The resulting structure can be considered a shortened penis lacking the glans and prepuce. However, it was accompanied by a bulbospongiosus muscle and penile retractor muscle.

It is known that freemartinism is a major type of bovine DSD, however, our study showed that other DSD forms can also alter the reproductive system of cows. The question to be answered is whether the abnormality has hereditary form, or occurs de novo. It is commonly accepted that cytogenetic examination is the first step in DSD diagnosis [2]. Usually, this is performed on in vitro cultured leukocytes and focuses on the constitution of the sex chromosome. In the heifer examined here, the vast majority of metaphases, originated from in vitro cultured leukocytes, had a normal female set of sex chromosomes (60,XX), but several appeared to be triploid (90,XXY). We thus decided to perform additional analysis of in vitro cultured fibroblasts derived from a skin sample. The presence of the triploid cell line, with an elevated frequency, was confirmed.

**Figure 6:**
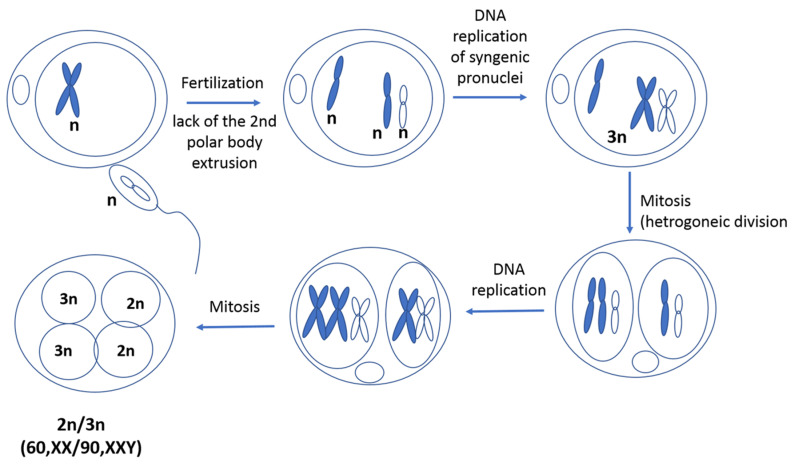
Possible mechanism of origin for 2n/3n mixoploidy in the heifer studied here.

The identification of two cell lines raised the question of whether we were dealing with mosaicism or chimerism. Analysis of sixteen microsatellite markers commonly used in parentage control showed that both cell lines originated from a single zygote; we therefore assumed that this heifer represented a case of diploid/triploid (60,XX/90,XXY) mosaicism. We suggest that the following events were responsible for this abnormality: (i) lack of extrusion of the second polar body, (ii) DNA replication and syngamy of male and female pronuclei, and (iii) postzygotic mitotic division accompanied by a random segregation of the nonreplicated unextruded second polar body, leading to the formation of the diploid and triploid blastomeres (Figure 6). This mechanism can be classified as asymmetric zygotic segregation of parental genomes [12] or heterogoneic cell division [18].

Several cases of diploid/triploid mixoploidy have been described in domestic mammals, specifically in cattle, cats, horses, and mink (Table 2). An XX diploid cell line and XXY triploid line were observed in almost all of them. Two features appeared to be common to these cases: the first was the absence of, or very low frequency of, a triploid cell line in leukocytes and its presence in fibroblasts, derived from different organs. Secondly, in all these cases disorders of sex development were noted.

Since routine chromosome analysis is carried out on in vitro cultured leukocytes, it can be suggested that the incidence of 2n,XX/3n,XXY mixoploidy may be underestimated, due to the very low incidence of the triploid cell line in leukocytes. We demonstrated that the use of highly sensitive ddPCR for the analysis of X-linked and Y-linked genes in DNA isolated from other tissues (skin, fibroblast culture, buccal epithelial cells, and hair follicles) facilitates the simple and reliable detection of Y-linked genes. We earlier demonstrated that this technique is also very useful in the detection of leukocyte chimerism in cattle [4] and pig [20] freemartins. In these studies, the *AMELY*/*AMELX* ratio was analyzed and it allowed the detection of the XY cell line, even if it occurred with a low frequency.

## 5. Conclusions

Our study confirmed that disorders of sex development in cattle can be caused by diploid/triploid mixoploidy (60,XX/90,XXY). This condition seems to be quite rare, but the cytogenetic analysis routinely performed on in vitro cultured leukocytes may underestimate its incidence. It is recommended, therefore, that different cell types—such as fibroblasts and hair follicles—should be examined in DSD cases. We have shown for the first time that the highly sensitive ddPCR technique for detecting low-level cell lines carrying the Y chromosome is very useful in such studies.

## Figures and Tables

**Table 1:** Summary of cytogenetic and molecular studies of the disorder of sex development (DSD) heifer.

**Cytogenetic Analysis**				
Tissue	Number of metaphase spreads			
	Total	60,XX	90,XXY	
Lymphocytes	400	388	12	
Fibroblasts	200	130	70	
**Molecular analysis**				
	PCR		ddPCR	
	*SRY*		*AMELX*	*AMELY*
Blood	+		+	+
Skin	+		+	+
Fibroblasts	+		+	+
buccal epithelial cells	+		+	+
hair follicles	not detectable		+	+

**Table 2:** An overview of mixoploidy cases diagnosed postnatally in domestic animals.

Species	Karyotype	Information about Frequency of Triploid Metaphases in Different Tissues	Phenotype	Reference
Cattle	60,XX/90,XXY	very low in leukocytes and elevated in fibroblasts (different organs)	Penis, empty scrotum, ovary with corpus luteum, ovotestis with epididymis; ductus deferens; uterus	[6]
	60,XX/90,XXY	absent from leukocytes and bone marrow, present in fibroblasts	aplasia of vulva, rudimentary penis, ovaries	[7]
	60,XX/90,XXY		aplasia of vulva, rudimentary penis	[7]
	60,XX/90,XXY	absent from leukocytes, elevated in fibroblasts	aplastic vulva, penis and clitoris agenesis, a male-like urethra located in a pseudoprepuce opening between the mammary complexes, cycling ovaries	[8]
	60,XX/90,XXY	low in leukocytes, elevated in fibroblasts	deformed vulva, blind vagina, extra urethra included in the shortened penis with sigmoid flexure; presence of bulbospongiosus muscle and retractor muscle of penis	This study
Cat	38,XX/57,XXY	not studied in leukocytes, present in fibroblasts (ear)	tortoiseshell male cat with spermatogenetically inactive testes	[9]
	38,XX/57,XXY	absent from leukocytes and low in fibroblasts (skin)	rudimentary penis, female genital tract, hypoplastic testis	[10]
	38,XX/57,XXY	not studied in leukocytes, present in fibroblasts (testes)	tortoiseshell male cat with spermatogenetically inactive testes	
	38,XY/57,XXY	not studied in leukocytes, low in fibroblasts (skin)	tortoiseshell male cat with spermatogenetically active testes	
Horse	64,XX/96,XXY	absent from leukocytes and very high in fibroblasts (skin)	enlarged clitoris, with partially sealed vulva lips, urinary tract orifice in a vestigial vagina	[11]
Mink	30,XX/45,XXY	not studied in leukocytes, elevated in fibroblasts derived from different organs	enlarged clitoris with os penis, uterus, ovotestes	[19]

## Data Availability

The data presented in this study are available on request from the corresponding author.

## Supplementary Materials

The following are available online at https://www.mdpi.com/2076-2615/11/2/285/s1, Figure S1: PCR detection of the *SRY* gene (813 bp). L: DNA ladder; 1: blood; 2: hair follicles (not detected); 3: buccal epithelial cells (a weak band is visible); 4: skin; 5: fibroblasts; 6: male reference; 7: female reference; 8: negative control (sample with no DNA template). Table S1: genotypes (length of the fragments in base pairs) at sixteen microsatellite loci in the DSD heifer and her sire.

## References

[1] Parma P., Veyrunes F., Pailhoux E. (2016). Sex reversal in non-human placental mammals. Sex. Dev..

[2] Szczerbal I., Switonski M., Payan-Careira R. (2016). Chromosome Abnormalities in Domestic Animals as Causes of Disorders of Sex Development or Impaired Fertility. Insights from Animal Reproduction.

[3] Raudsepp T., Chowdhary B.P. (2016). Chromosome aberrations and fertility disorders in domestic animals. Annu. Rev. Anim. Biosci..

[4] Szczerbal I., Nowacka-Woszuk J., Albarella S., Switonski M. (2019). Technical note: Droplet digital PCR as a new molecular method for a simple and reliable diagnosis of freemartinism in cattle. J. Dairy Sci..

[5] Kozubska-Sobocińska A., Smołucha G., Danielak-Czech B. (2019). Early diagnostics of freemartinism in Polish Holstein-Friesian female calves. Animals.

[6] Dunn H.O., McEntee K., Hansel W. (1970). Diploid-triploid chimerism in a bovine true hermaphrodite. Cytogenet. Genome Res..

[7] Rieck G.W., Höhn H., Schmidt I. (1982). Vulvaaplasie und urethra masculina: Maskulinisierungseffekte des sinus urogenitalis bei genetisch weiblichen rindern durch chimärismen mit dem XXY-gonosomen-komplement [Aplastic vulva and masculine urethra: Masculinization effects of the urogenital sinus in genetically female cattle due to chimerism with the XXY gonosomal complement]. Berl. Munch. Tierarztl. Wochenschr..

[8] Meinecke B., Drogemuller C., Kuiper H., Burstel D., Wohlsein P., Ebeling S., Wehrend A., Meinecke-Tillmann S. (2007). A diploid-triploid (60, XX/90, XXY) intersex in a Holstein heifer. Sex. Dev..

[9] Chu E.H., Thuline H.C., Norby D.E. (1964). Triploid-diploid chimerism in a male tortoiseshell cat. Cytogenet. Genome Res..

[10] Gregson N.M., Ishmael J. (1971). Diploid-triploid chimerism in 3 tortoiseshell cats. Res. Vet. Sci..

[11] Power M.M., Leadon D.P. (1990). Diploid-triploid chimaerism (64, XX/96, XXY) in an intersex foal. Equine Vet. J..

[12] Carson J.C., Hoffner L., Conlin L., Parks W.T., Fisher R.A., Spinner N., Yatsenko S.A., Bonadio J., Surti U. (2018). Diploid/triploid mixoploidy: A consequence of asymmetric zygotic segregation of parental genomes. Am. J. Med. Genet. Part A.

[13] Van de Laar I., Rabelink G., Hochstenbach R., Tuerlings J., Hoogeboom J., Giltay J. (2002). Diploid/triploid mosaicism in dysmorphic patients. Clin. Genet..

[14] Boonen S.E., Hoffmann A.L., Donnai D., Tümer Z., Ravn K. (2011). Diploid/triploid mosaicism: A rare event or an under-diagnosed syndrome?. Eur. J. Med. Genet..

[15] Szczerbal I., Kociucka B., Nowacka-Woszuk J., Lach Z., Jaskowski J.M., Switonski M. (2014). A high incidence of leukocyte chimerism (60, XX/60, XY) in single born heifers culled due to underdevelopment of internal reproductive tracts. Czech. J. Anim. Sci..

[16] Padula A.M. (2005). The freemartin syndrome: An update. Anim. Reprod. Sci..

[17] Peretti V., Ciotola F., Albarella S., Paciello O., Dario C., Barbieri V., Iannuzzi L. (2008). XX/XY chimerism in cattle: Clinical and cytogenetic studies. Sex. Dev..

[18] Destouni A., Zamani Esteki M., Catteeuw M., Tšuiko O., Dimitriadou E., Smits K., Kurg A., Salumets A., Van Soom A., Voet T. (2016). Zygotes segregate entire parental genomes in distinct blastomere lineages causing cleavage-stage chimerism and mixoploidy. Genome. Res..

[19] Nes N. (1966). Diploid-triploid chimerism in a true hermaphrodite mink (Mustela vison). Hereditas.

[20] Szczerbal I., Nowacka-Woszuk J., Dzimira S., Matuszczyk A., Iskrzak P., Switonski M. (2019). Elevated incidence of freemartinism in pigs detected by droplet digital PCR and cytogenetic techniques. Livest. Sci..

