# Supplementary material for: A Disorder of Sex Development in a Holstein–Friesian Heifer with a Rare Mosaicism (60,XX/90,XXY): A Genetic, Anatomical, and Histological Study
Source: Animals (Basel). 2021 Jan 23;11(2):285. doi: 10.3390/ani11020285 (PMC7911242; doi:10.3390/ani11020285)
Supplement: Supplementary file 1 [file animals-11-00285-s001.pdf]

animals

Supplementary Material

A Disorder of Sex Development in a Holstein–Friesian Heifer with a Rare Mosaicism (60,XX/90,XXY): A Genetic, Anatomical, and Histological Study

Izabela Szczerbal [1], Marcin Komosa [2], Joanna Nowacka-Woszuk [1], Tomasz Uzar [2], Marek Houszka [3], Jerzy Semrau [4], Magdalena Musial [4], Michal Barczykowski [4], Anna Lukomska [3] and Marek Switonski [1,*]

[1] Department of Genetics and Animal Breeding, Poznan University of Life Sciences, 60-637 Poznan, Poland; izabela.szczerbal@up.poznan.pl (I.S.); joanna.nowacka-woszuk@up.poznan.pl (J.N.-W.)
[2] Department of Animal Anatomy, Poznan University of Life Sciences, 60-625 Poznan, Poland; marcin.komosa@up.poznan.pl (M.K.); tomasz.uzar@up.poznan.pl (T.U.)
[3] Department of Preclinical Sciences and Infectious Diseases, Poznan University of Life Sciences, 60-637 Poznan, Poland; marek.houszka@up.poznan.pl (M.H.); anna.lukomska@up.poznan.pl (A.L.)
[4] Center for Animal Health and Reproduction, 88-200 Radziejow, Poland; jassemrau@gmail.com (J.S.); madzix.es@gmail.com (M.M.); m.barczykowski@op.pl (M.B.)
* Correspondence: marek.switonski@up.poznan.pl

Citation: Szczerbal, I.; Komosa, M.; Nowacka-Woszuk, J.; Uzar, T.; Houszka, M.; Semrau, J.; Musial, M.; Barczykowski, M.; Lukomska, A.; Switonski, M. A Disorder of Sex Development in a Holstein–Friesian Heifer with a Rare Mosaicism (60,XX/90,XXY): A Genetic, Anatomical, and Histological Study. *Animals* **2021**, *11*, 285. https://doi.org/10.3390/ani11020285

Academic Editor: Francesca Ciotola, Sara Albarella, Vincenzo Peretti
Received: 21 December 2020
Accepted: 20 January 2021
Published: 23 January 2021

Publisher's Note: MDPI stays neutral with regard to jurisdictional claims in published maps and institutional affiliations.

Copyright: © 2021 by the authors. Licensee MDPI, Basel, Switzerland. This article is an open access article distributed under the terms and conditions of the Creative Commons Attribution (CC BY) license (http://creativecommons.org/licenses/by/4.0/).

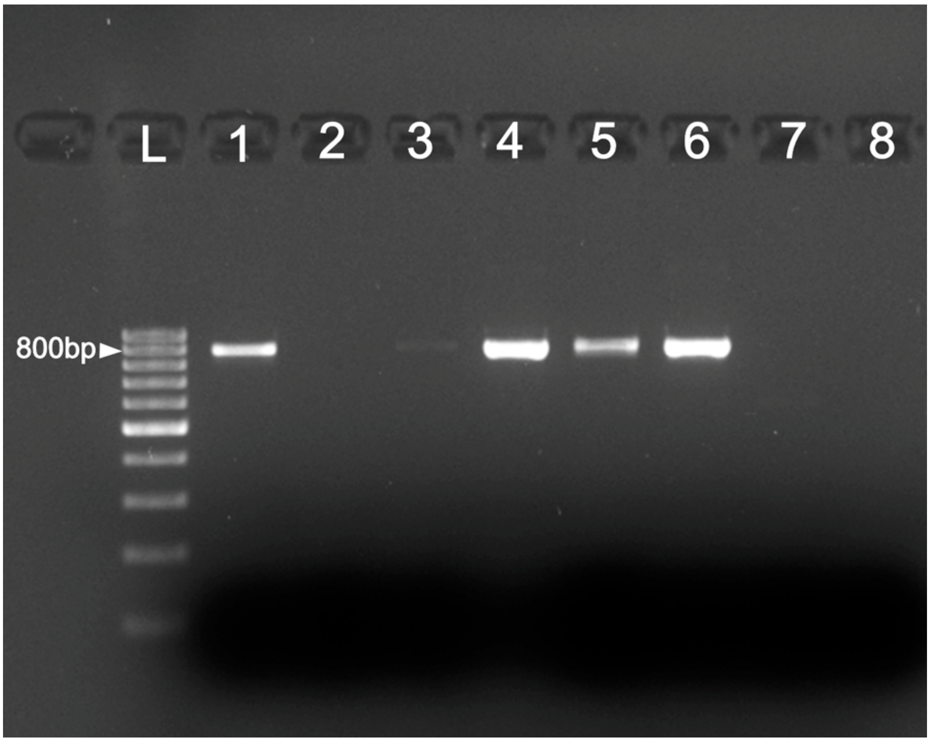

Figure S1. PCR-detection of the *SRY* gene (813 bp). L: DNA ladder; 1: blood; 2: hair follicles (not detected); 3: buccal epithelial cells (a weak band is visible); 4: skin; 5: fibroblasts; 6: male reference; 7: female reference; 8: negative control (sample with no DNA template).

Table S1. Genotypes (length of the fragments in base pairs) at 16 microsatellite *loci* in the DSD heifer and her sire.

Marker	Sire	DSD Heifer	
		Fibroblasts	Hair Follicles
TGLA227	91/97	89/91	89/91
BM2113	125/127	125/127	125/127
ETH10	219/219	217/219	217/219
SPS115	248/260	260/260	260/260
SPS113	141/151	141/147	141/147
RM067	90/92	92/92	92/92
TGLA126	115/117	117/117	117/117
TGLA122	163/183	163/171	163/171
INRA23	202/206	206/206	206/206
BM1818	262/266	262/266	262/266
ETH225	144/150	140/150	140/150
BM1824	188/188	188/188	188/188
CSRM60	92/98	98/102	98/102
MGTG4B	141/141	141/141	141/141
CSSM66	183/183	183/183	183/183
ILSTS006	288/292	288/288	288/288
